# Difference between physical therapist estimation and psychological patient-reported outcome measures in patients with low back pain

**DOI:** 10.1371/journal.pone.0227999

**Published:** 2020-01-21

**Authors:** Takahiro Miki, Yu Kondo, Tsuneo Takebayashi, Hiroshi Takasaki

**Affiliations:** 1 Department of Rehabilitation, Sapporo Maruyama Orthopedic Hospital, Sapporo, Hokkaido, Japan; 2 Department of Orthopedic, Sapporo Maruyama Orthopedic Hospital, Sapporo, Hokkaido, Japan; 3 Department of Physical Therapy, Saitama Prefectural University, Koshigaya, Saitama, Japan; Medical University Innsbruck, AUSTRIA

## Abstract

Minimizing the number of patient-reported outcome measures (PROMs) can reduce patient burden. The primary aim of the present study was to investigate whether physical therapists (PTs) can estimate psychological PROM scores in patients with low back pain (LBP) through physical therapy evaluation. The secondary aims were; 1) to investigate whether the clinical experiences of PTs influence correlations between PT estimates and psychological PROM scores, and 2) to investigate the sensitivity and specificity of PT estimates for the psychological features detected by the PROMs. We recruited hospitalized patients owing to LBP, who underwent evaluation by PTs on the initial day of hospitalization. Patients completed PROMs, including the Pain Catastrophizing Scale (PCS), Tampa Scale for Kinesiophobia, and Hospital Anxiety and Depression Scale immediately before the initial physical therapy session. PTs rated the magnitude of patient kinesiophobia, pain catastrophizing, anxiety, and depression using an 11-point numerical rating scale (NRS; 0 = not detected at all, 10 = very highly detected) through physical therapy evaluation immediately after the initial session. The PTs were blinded to the PROM results. We categorized PTs into two subgroups (PTs with ≥4 years and those with <4 years of clinical experience). Data from 78 patients (mean [SD] age = 60.5 [16.3] years) and 21 PTs were analyzed. A statistically significant but weak correlation (*P* = .04, Spearman’s *ρ* = .24) was detected only in the total PCS scores and PT NRS scores in a dataset of all patients and PTs. Further, there were no statistically significant differences in correlations (all *P* >.05) between the two subgroups of PTs in all measures. Low sensitivity and high specificity of PT estimates for psychological features through physical therapy evaluation were identified in all PROMs when PT NRS scores were categorized into the binary score by 5 (negative: <5; positive: ≥5).

## Introduction

Low back pain (LBP) causes substantial burden globally, and the disability level and prognosis of LBP are known to be associated with psychological features, including kinesiophobia, pain catastrophizing, anxiety, and depression [1,2,3]. Thus, it is important for physical therapists (PTs) to understand a patient’s magnitude of psychological deficits in order to undertake LBP management from biopsychosocial perspectives.

Minimizing the number of patient-reported outcome measures (PROMs) can reduce patient burden [4]. It is reported that general practitioners have low sensitivity with regard to the identification of kinesiophobia and pain catastrophizing in patients with acute LBP. However, a previous study reported the possibility of identifying the characteristics of patients with increased kinesiophobia through clinical gait assessment [5]. Therefore, it can be hypothesized that PTs, who are experts in movement evaluation, may be able to estimate similar features of psychological deficits through physical therapy evaluation including clinical interview/ subjective and physical assessments.

The clinical experiences of PTs can be a confounding factor of the ability to estimate some psychological features through physical therapy evaluation [6]. It is demonstrated that the clinical experiences of PTs influenced the accuracy of PT estimates for the patient anxiety rating scale in the 10-item Örebro Musculoskeletal Pain Screening Questionnaire (OMSQ) [6]. However, this study [6] had limitations as psychological deficits were not evaluated with established measures and cutoff values were not considered. Therefore, a new study involving PROMs that are commonly used in research and clinical practice with cutoff scores is required to further understand whether the clinical experiences of PTs influence the accuracy of PT estimates for the psychological PROM scores in patients with LBP through physical therapy evaluation. Further, it would be beneficial to understand the sensitivity and specificity of PT estimates for the psychological status through physical therapy evaluation by considering the cutoff values in the PROMs in order to consider strategies to minimize patient burden associated with responding to many PROMs.

The primary aim of the present study was to investigate whether PTs can estimate psychological PROM scores, including kinesiophobia, pain catastrophizing, anxiety, and depression, in patients with LBP through physical therapy evaluation. The secondary aims were; 1) to investigate whether the clinical experiences of PTs influence correlations between PT estimates and psychological PROM scores, and 2) to investigate the sensitivity and specificity of PT estimates for the psychological status detected by the PROMs.

## Material and methods

### Design

This cross-sectional study was performed at a single hospital. The institutional research ethics committee (Sapporo Maruyama Orthopedic Hospital, #000016) approved the study protocol. All participants and PTs provided written consent before data collection.

### Participants

Using convenience sampling, we recruited patients with LBP from an orthopedic hospital in Hokkaido, Japan (Sapporo Maruyama Orthopedic Hospital). The inclusion criteria were; 1) in-patient hospital stay for the LBP and/or low back related leg pain with a medical diagnosis of lumbar canal stenosis or lumbar disk herniation between January 2018 and March 2019; 2) age 20–90 years; 3) no use of painkillers at the initial physical therapy session; 4) physical therapy evaluations by PTs on the initial day of hospitalization; 5) no serious pathologies, including pyogenic spondylitis and cauda equina syndrome; and 6) no history of neurological disorders or heart diseases.

### Physical therapists

PTs seeing patients with LBP at least once a week were recruited during the research period. Two authors (TM and YK) explained the study aim and background and provided information about the used PROMs and corresponding psychometric properties to the PTs participating in the present study.

### Procedures

Participants completed all of the following PROMs immediately before the initial physical therapy session: 1) 11-point numerical rating scale (NRS) for pain intensity [7–9]; 2) Roland Morris Disability Questionnaire (RMDQ) for disability due to LBP [10]; 3) Euro QOL 5 Dimensions (EQ-5D) for quality of life [11]; 4) Pain Catastrophizing Scale (PCS) for pain catastrophizing [12]; 5) Tampa Scale for Kinesiophobia (TSK) for kinesiophobia [13]; and 6) Hospital Anxiety and Depression Scale (HADS) for anxiety and depression [14]. Participant age, sex, height, weight, and medical diagnosis were collected from medical records. PTs rated the magnitude of kinesiophobia, pain catastrophizing, anxiety, and depression using an 11-point NRS (0 = not detected at all, 10 = very highly detected) immediately after the initial physical therapy session. The PTs were blinded to the results of the PROMs.

The initial physical therapy session, which lasted 40 minutes, included; 1) the subjective assessment to understand the level of pain, the symptoms, and the patient’s goals for the physical therapy; 2) the objective assessment, excluding the PROMs, to evaluate alignment, mobility, strength, endurance, and functional movements and neurological examinations when necessary; and 3) the setting of goals. There was no standardization among the PTs in the forms of physical therapy evaluation and treatment used in this study.

#### Eleven-point numerical rating scale for pain intensity

We used an 11-point NRS to determine the average pain intensity on the day of the evaluation of the lower back and lower extremities. A score of 0 indicates no pain, and a score of 10 indicates the worst imaginable pain.

#### Roland Morris disability questionnaire

We used the Japanese version of the RMDQ [15]. The RMDQ includes 24 items with a dichotomous scale (No = 0, Yes = 1). A higher total score indicates greater disability due to LBP. The concurrent validity, high internal consistency (Cronbach alpha of all items = .86), and high test–retest reliability (.95) of the Japanese version of the RMDQ have been confirmed [15].

#### Euro QOL 5 dimensions

We used the Japanese version of the EQ-5D [16], which is a commonly used measure for quality of life and has five items with five categorical scales. A higher score indicates better quality of life, with a maximum score of one [17].

#### Pain Catastrophizing Scale

We used the Japanese version of the PCS [18, 19]. The PCS includes 13 items, with scores ranging from 0 to 4. A higher total score indicates a greater deficit in pain catastrophizing, and a total score of 30 is the cutoff point for clinically relevant levels of catastrophizing [12]. The construct validity and high internal consistency (Cronbach alpha of all items = .89) of the Japanese versions of the PCS were confirmed [19].

#### Tampa scale for kinesiophobia

We used the Japanese version of the TSK [20]. The TSK includes 17 items, with scores ranging from 1 to 4. A higher total score indicates greater kinesiophobia, and a total score of 37 is the cutoff point for strong kinesiophobia [20] . The concurrent validity and high internal consistency (Cronbach alpha of all items = .85) of the Japanese version of the TSK have been confirmed [20].

#### Hospital anxiety and depression scale

We used the Japanese version of the HADS [21]. The HADS includes 14 items, with scores ranging from 0 to 3. Odd number items are for depression and even number items are for anxiety. A higher total score indicates greater anxiety or depression. In each subscale of anxiety or depression, a total score of 8 is the cutoff point for the identification of suspicious cases for depression and anxiety [21]. The construct validity of this scale has been confirmed [21].

### Statistical analysis

Descriptive analyses were used to understand the characteristics of both the participants and the PTs. The education levels of the PTs were categorized into diploma, bachelor’s degree, master’s degree and doctorate’s degree. The entry level education of a Japanese PT includes a diploma (minimum educational level, 3–4 years) and bachelor’s degree (4 years). For convenience, we calculated Spearman’s ρ values between the total patient scores of the PCS, TSK, and depression and anxiety subscales of the HADS and the PT NRS scores and between the binary patient scores of the PCS, TSK, and depression and anxiety subscales of the HADS with established cutoff values (i.e., ≥ or < the cutoff value; 0 = negative, 1 = positive) and the PT NRS scores. In order to investigate whether the clinical experiences of PTs influence correlations between the total and binary psychosocial PROM scores and the corresponding PT NRS scores, we categorized PTs into two subgroups (PTs with ≥4 years and those with <4 years of clinical experience, as 4-year clinical experience is necessary to supervise students with regard to clinical placement in Japan). We compared correlations between the two subgroups using independent correlation comparisons performed with Simple Interactive Statistical Analysis online software (www.quantitativeskills.com/sisa/statistics/corrhlp.htm, accessed June 13, 2019), similar to the approach in a previous study [6]. In the present study, ρ values of ≤ .40, .40–.75, and ≥ .75 were considered to indicate weak or no correlation, moderate correlation, and strong correlation, respectively [22]. Spearman’s ρ values were calculated using JMP Pro14^®^ (SAS Institute Inc., Cary, NC, USA). The alpha level was set at 5%.

For sensitivity and specificity analyzes, we evaluated PT NRS scores cutoff by investigating the Youden Index in the Receiver operating characteristic curve with the use of the binary PROM scores. The sensitivity and specificity analyzes were undertaken in all PTs and in the two PT subgroups, respectively.

## Results

Seventy eight patients (50 patients with lumbar canal stenosis and 28 patients with lumbar disk herniation, mean [SD] age = 60.5 [16.3] years) and 21 PTs participated in the current study. The demographics of the 78 patients and 21 PTs are summarized in Table 1. The two PTs with the doctor’s degree or the master’s degree did not complete a post-graduate clinical training program.

**Table 1:** Characteristics of the 78 patients and 21 physical therapists.

Patients	
Age (*yr*), mean (SD)	60.5 (16.3)
Gender (*n of men*), (%)	55 (70.5)
Body Mass Index (*kg/m*^*2*^), mean (SD)	27.4 (23.2)
Pain intensity over the lower back (*0–10*), mean (SD)	4.6 (2.7)
Pain intensity over the lower limb (*0–10*), mean (SD)	5.8 (2.7)
Roland-Morris Disability Questionnaire (*0–24*), mean (SD)	10.9 (5.6)
Euro QOL 5 Dimensions (*0–1*), mean (SD)	0.57 (0.16)
Pain Catastrophizing Scale (*0–52*), mean (SD)	28.4 (11.4)
Tampa Scale for Kinesiophobia (*17–68*), mean (SD)	40.3 (7.9)
Hospital Anxiety and Depression Scale-anxiety subscale (0*–21*), mean (SD)	5.4 (3.6)
Hospital Anxiety and Depression Scale-depression subscale (0*–21*), mean (SD)	5.5 (3.4)
**Physical Therapists**	
Age (*yr*), mean (SD)	29.4 (4.9)
Gender (*n of men*), (%)	16 (76.2)
Years after acquisition of a physical therapist license (*yr*), mean (SD)	7.0 (4.7)
Physical therapists with ≥4 years of clinical experience (*n*), %	15 (71.5)
Education level	
Doctor’s degree (*n*), (%)	1 (4.7)
Master’s degree (*n*), (%)	1 (4.7)
Bachelor’s degree (*n*), (%)	4 (19.0)
Diploma (*n*), (%)	15 (71.5)

Table 2 presents the correlations among total and binary psychological PROM scores and NRS scores in all PTs, where details are provided in Appendix (S1 Appendix), and comparisons of correlations between the two subgroups of PTs, where details are provided in Appendix (S2 Appendix). With regard to the data of all PTs, a statistically significant (*P* = .04) correlation was detected only in the total PCS scores. In a subgroup of PTs with clinical experience ≥4 years, statistically significant correlations were detected only in the total PCS scores (*P* = .007) and in the binary PCS scores (*P* = .04). However, there were no statistically significant differences (all *P* >.05) between the two subgroups of PTs in all measures.

**Table 2:** Correlations (Spearman’s *ρ*) among total and binary scores of psychological patient-reported outcome measure scores and physical therapist (PT) numerical rating scales in all PTs and comparisons of correlations between the two subgroups of PTs according to clinical experience.

Variables	Correlation analysis	Comparisons of correlations		
	All PTs (n = 21)	Clinical experience ≥4 years (15 PTs and 53 patients)	Clinical experience <4 years (6 PTs and 25 patients)	Z-value (*P*)
PCS	.24*	.37^†^	.06	1.28 (.20)
Binary PCS	.19	.31*	.03	1.1 (.26)
TSK	.10	.05	.19	0.56 (.58)
Binary TSK	.17	.12	.25	0.52 (.60)
HADS-A	.15	.13	.32	0.79 (.43)
Binary HADS-A	.17	.26	-.01	1.08 (.28)
HADS-D	.12	.21	-.02	0.91 (.36)
Binary HADS-D	< .001	.10	-.16	1.02 (.31)

*P < .05

^†^*P* < .01

Abbreviations: PCS, total score in the Pain Catastrophizing Scale; binary PCS, binary score in the PCS; TSK, total score in the Tampa Scale for Kinesiophobia; binary TSK, binary score in the TSK; HADS-A, total score in the Hospital Anxiety and Depression Scale for anxiety; binary HADS-A, binary score in the HADS-A; HADS-D, total score in the Hospital Anxiety and Depression Scale for depression; binary HADS-D, binary score in the HADS-D.

Table 3 presents the sensitivity and specificity of PT estimates for psychological features through physical therapy evaluation considering the binary PROM scores in all PTs and in the two PT subgroups. The calculated cutoff points by the Youden Index were less than 5 in all measures and mostly 3.

**Table 3:** Sensitivity and specificity of physical therapist (PT) estimates for psychological features through physical therapy evaluation considering the binary patient-reported outcome measure scores in all PTs and in the two PT subgroups according to clinical experience.

NRS threshold*	All PTs (78 patients and 21 PTs)				PTs with clinical experience ≥4 yeas (53 patients and 15 PTs)				PTs with clinical experience <4 years (25 patients and 6 PTs)			
	PCS	TSK	HADS-A	HADS-D	PCS	TSK	HADS-A	HADS-D	PCS	TSK	HADS-A	HADS-D
0/1-10	1/0	1/0	1/0	1/0	1/0	1/0	1/0	1/0	1/0	1/0	.33/.89^†^	.63/.59^†^
0-1/2-10	.76/.27	.85/.20	.81/.18	.55/.32	.86/.23	.89/.18	.87/.21	.64/.28	.60/.40	.76/.25	.33/89	.75/.41
0-2/3-10	.60/.59	.70/.48	.71/.39	.50/.59	.68/.61	.69/.35	.73/.37	.64/.59^†^	.47/.50	.71/.75^†^	.33/58	.75/.18
0-3/4-10	.46/.80^†^	.53/.68^†^	.67/.58^†^	.32/.79^†^	.59/.77^†^	.53/.65^†^	.73/.53	.36/.77	-	.53/.75	.50/.32	-
0-4/5-10	.35/.88	.32/.76	.43/.74	.18/.88	.40/.87	.33/.71	.53/.74^†^	.14/.90	.27/.90^†^	.29/.88	.83/.26	.88/.12
0-5/6-10	.21/.90	.25/88	.33/.82	.09/.95	.23/.90	.25/.88	.40/.84	.07/.97	.20/.90	.24/.88	.83/.11	-
0-6/7-10	.05/.93	.17/.88	.24/.91	-	.05/.93	.17/.88	.27/.92	-	-	.18/.88	-	-
0-7/8-10	.05/.98	.11/.88	.24/.95	.09/.96	-	.11/.88	.27/.97	-	.07/.90	.12/.88	1/.11	1/.12
0-8/9-10	.03/.98	.08/.96	.05/.96	.05/.96	.05/1	.08/.94	.07/1	.07/1	-	-	1/0	1/.06
0-9/10	0/.98	.02/1	-	0/.98	-	-	-	-	0/.90	.06/1	-	1/0

*Negative/positive

^†^NRS cutoff point detected by the Youden Index

Values are sensitivity/specificity.

Abbreviations: NRS, physical therapists rating numerical rating scale; PCS, Pain Catastrophizing Scale; TSK, Tampa Scale for Kinesiophobia; HADS-A, Hospital Anxiety and Depression Scale for anxiety; HADS-D, Hospital Anxiety and Depression Scale for depression; -, no data.

## Discussion

This study investigated whether PTs can estimate the psychological PROM scores, including kinesiophobia, pain catastrophizing, anxiety, and depression, in patients with LBP through physical therapy evaluation. A statistically significant correlation was detected only in the total PCS scores and PT NRS scores. However, the correlation was weak and there was no statistically significant correlation in binary score of the PCS. These results indicate that it is difficult for PTs to estimate the psychological PROM scores through physical therapy evaluation, and this is consistent with previous findings [4, 6, 23, 24].

We investigated whether the clinical experiences of PTs influence correlations between PT estimates and psychological PROM scores. There was no difference in any correlation between PTs with clinical experience ≥4 years and those with clinical experience <4 years. These results differ from the findings in a previous study that included Australian PTs [6]. These differences might have resulted from potential differences in the clinical skills of PTs between Australia and Japan, which can be assumed by limited use of an evidence database and an attitude that focuses more on biomedical perspectives than biopsychosocial perspectives among Japanese PTs [25, 26] and limited practice permitted to Japanese PTs [27]. Further, there was no PT who completed a post-graduate clinical training program. The outcome measure might have also influenced the different findings between the current study and the previous study [6]. In the previous study [6], the researchers used an item from the 10-item OMSQ for the degree of patient anxiety, which was an 11-point NRS for anxiety. There might have been high correlation between the therapist estimate and the patient-reported outcome because the same scale was used. However, in the present study, structured PROMs with multiple questions were used. Our results suggest that increased clinical experience only cannot enhance the ability to estimate psychological PROM scores through physical therapy evaluation. It would be required to investigate whether certain post-graduate clinical training programs might affect the ability to estimate psychological PROM scores through physical therapy evaluation.

Regarding the sensitivity and specificity analyses, the calculated cutoff points by the Youden Index were less than 5 in all measures and mostly 3. These findings indicate that the PTs tended to underestimate the presence of patients’ psychological problems. Low sensitivity and high specificity were noted when the NRS scores were categorized into the binary score by 5 (negative: < 5; positive: ≥ 5). This finding indicates the inability of PTs to screen patients with psychological problems through physical therapy evaluation and suggest the benefit of using a screening tool for psychological status.

### Clinical Implications and Research Agenda

The results of the present study suggest the use of screening questionnaires to compensate for the low sensitivity of PT identification of psychological problems among patients. Some screening questionnaires for LBP prognosis have been proposed [28, 29]. However, no study has shown the Japanese cutoff points for screening questionnaires that have high sensitivity to detect psychological problems. Thus, future studies are warranted.

### Limitations

The present study has three potential limitations. The first limitation is that the samples were limited, considering the limited medical diagnoses, hospitalized patients, dominant leg pain, mean age of 60 years, 70% of male patients, and the Japanese population (i.e., potential influence of cultural aspects). These factors might have affected PTs’ estimate of patients’ psychological status. The second limitation is that there was no standardization in the forms of physical therapy evaluation and treatment used. In addition, the majority of the PTs were only diploma holders, and none of the PTs had completed a post-graduate clinical training program. Thus, there is a potential limitation in the generalizability of the current findings. Although the sample size in the present study is similar to that in previous studies [6, 30], larger multi-center cohorts will be required to increase generalizability. Third, we did not investigate PT’s knowledge about pain neuroscience [31], their attitude toward biopsychosocial perspectives for LBP management [24, 32, 33], and their adherence to clinical practice guidelines for LBP management [34]. These potential confounders should be considered in future studies with larger samples of PTs to help identify the contributing factors for increasing the ability of PTs to understand a patient’s psychological status through physical therapy evaluation.

## Conclusions

The present study found that PTs cannot estimate the psychological PROM scores, including kinesiophobia, pain catastrophizing, anxiety, and depression, and that clinical experience does not influence the accuracy of PT estimates in patients with LBP through physical therapy evaluation. Additionally, PTs show low sensitivity and high specificity for the identification of the psychological problems through physical therapy evaluation.

## Supporting information

S1 Appendix Correlations among total and binary scores of psychological patient-reported outcome measure scores and physical therapist (PT) numerical rating scales in all PTs. (DOCX)

S2 Appendix Correlations among total and binary scores of psychological patient-reported outcome measure scores and physical therapist (PT) numerical rating scales in two subgroups of PTs based on clinical experience. (DOCX)

S3 Appendix Dataset in the current study. (XLSX)
