# Supplementary material for: Difference between physical therapist estimation and psychological patient-reported outcome measures in patients with low back pain
Source: PLoS One. 2020 Jan 21;15(1):e0227999. doi: 10.1371/journal.pone.0227999 (PMC6974035; doi:10.1371/journal.pone.0227999)
Supplement: S1 Appendix — (DOCX) [file pone.0227999.s001.docx]

S1 Appendix 1. Correlations among total and binary scores of psychological patient-reported outcome measure scores and physical therapist (PT) numerical rating scales in all PTs.

|  | 2 | 3 | 4 | 5 | 6 | 7 | 8 | 9 | 10 | 11 | 12 |
| --- | --- | --- | --- | --- | --- | --- | --- | --- | --- | --- | --- |
| 1. PCS | .21 | .47^‡^ | .42^‡^ | .24* | .07 | .14 | .16 |  |  |  |  |
| 1. TSK |  | .21 | .03 | .16 | .10 | .05 | .07 |  |  |  |  |
| 1. HADS-A |  |  | .49^‡^ | .28* | .09 | .15 | .12 |  |  |  |  |
| 1. HADS-D |  |  |  | .12 | .04 | .16 | .12 |  |  |  |  |
| 1. PT-PC |  |  |  |  | .66^‡^ | .68^‡^ | .72^‡^ | .19 | .20 | .24* | .09 |
| 1. PT-KF |  |  |  |  |  | .62^‡^ | .59^‡^ | .08 | .17 | .03 | .02 |
| 1. PT-A |  |  |  |  |  |  | .73^‡^ | .13 | .13 | .17 | .02 |
| 1. PT-D |  |  |  |  |  |  |  | .15 | .16 | .07 | <.001 |
| 1. binary PCS |  |  |  |  |  |  |  |  | .26* | .34^†^ | .26* |
| 1. binary TSK |  |  |  |  |  |  |  |  |  | .23* | <.001 |
| 1. binary HADS-A |  |  |  |  |  |  |  |  |  |  | .39^‡^ |
| 1. binary HADS-D |  |  |  |  |  |  |  |  |  |  |  |

**P* < .05

^†^*P* < .01

^‡^*P* < .001

Values are Spearman’s *ρ* values.

Abbreviations: PCS, total score in the Pain Catastrophizing Scale; TSK, total score in the Tampa Scale for Kinesiophobia; HADS-A, total score in the Hospital Anxiety and Depression Scale for anxiety; HADS-D, total score in the Hospital Anxiety and Depression Scale for depression; PT-PC, PT 11-point numerical rating scale for patient’s pain catastrophizing; PT-KF, PT 11-point numerical rating scale for patient’s kinesiophobia; PT-A, PT 11-point numerical rating scale for patient’s anxiety; PT-D, PT 11-point numerical rating scale for patient’s depression; binary PCS, binary score in the PCS; binary TSK, binary score in the TSK; binary HADS-A, binary score in the HADS-A; binary HADS-D, binary score in the HADS-D.
