# Supplementary material for: Difference between physical therapist estimation and psychological patient-reported outcome measures in patients with low back pain
Source: PLoS One. 2020 Jan 21;15(1):e0227999. doi: 10.1371/journal.pone.0227999 (PMC6974035; doi:10.1371/journal.pone.0227999)
Supplement: S2 Appendix — (DOCX) [file pone.0227999.s002.docx]

S2 Appendix 2. Correlations among total and binary scores of psychological patient-reported outcome measure scores and physical therapist (PT) numerical rating scales in two subgroups of PTs based on clinical experience.

|  | Clinical experience ≥4 yeas  (53 patients and 15 PTs) | | | | Clinical experience <4 years  (25 patients and 6 PTs) | | | |
| --- | --- | --- | --- | --- | --- | --- | --- | --- |
|  | 5 | 6 | 7 | 8 | 5 | 6 | 7 | 8 |
| 1. PCS | .37^†^ | .16 | .22 | .29* | .06 | -.04 | -.04 | -.03 |
| 1. TSK | .11 | .05 | .03 | -.05 | .30 | .19 | .13 | .40* |
| 1. HADS-A | .33^†^ | .08 | .13 | .16 | .30 | .13 | .32 | .11 |
| 1. HADS-D | .25 | .12 | .24 | .21 | -.07 | -.04 | .02 | -.02 |
| 1. PT-PC |  |  |  |  |  |  |  |  |
| 1. PT-KF | .59^‡^ |  |  |  | .80^‡^ |  |  |  |
| 1. PT-A | .62^‡^ | .52^‡^ |  |  | .76^‡^ | .80^‡^ |  |  |
| 1. PT-D | .70^‡^ | .47^‡^ | .73^‡^ |  | .71^‡^ | .76^‡^ | .66^‡^ |  |
| 1. Binary PCS | .31* | .18 | .21 | .34^†^ | .03 | -.07 | .01 | -.13 |
| 1. Binary TSK | .12 | .12 | .06 | .02 | .38 | .25 | .34 | .50* |
| 1. Binary HADS-A | .36^†^ | .12 | .26 | .18 | .02 | -.16 | -.01 | -.17 |
| 1. Binary HADS-D | .22 | .08 | .09 | .10 | -.14 | -.03 | -.13 | -.16 |

**P* < .05

^†^*P* < .01

^‡^*P* < .001

Values are Spearman’s *ρ* values.

Abbreviations: PCS, total score in the Pain Catastrophizing Scale; TSK, total score in the Tampa Scale for Kinesiophobia; HADS-A, total score in the Hospital Anxiety and Depression Scale for anxiety; HADS-D, total score in the Hospital Anxiety and Depression Scale for depression; PT-PC, PT 11-point numerical rating scale for patient’s pain catastrophizing; PT-KF, PT 11-point numerical rating scale for patient’s kinesiophobia; PT-A, PT 11-point numerical rating scale for patient’s anxiety; PT-D, PT 11-point numerical rating scale for patient’s depression; binary PCS, binary score in the PCS; binary TSK, binary score in the TSK; binary HADS-A, binary score in the HADS-A; binary HADS-D, binary score in the HADS-D.
